# Is the Calgary-Cambridge Model of consultation a suitable communication tool for students and newly qualified paramedics? A qualitative study

**DOI:** 10.29045/14784726.2024.6.9.1.23

**Published:** 2024-06-01

**Authors:** Claire Hastings

**Affiliations:** Sheffield Hallam University ORCID iD: https://orcid.org/0009-0003-4139-0981

**Keywords:** Calgary-Cambridge, communication, paramedic

## Abstract

**Introduction:**

The Calgary-Cambridge Model (C-CM), developed by Kurtz and Silverman in 1996, is a communication tool developed for doctors. Since its publication, it has been adopted by various healthcare professionals; however, no previous research has been identified that evaluates its use in paramedic practice. This study aims to explore the experience of students and newly qualified paramedics (NQPs) applying the C-CM in practice, and establish their experiences and perceptions of its suitability as a communication tool in the pre-hospital environment.

**Methods:**

This MSc research project, conducted in April–May 2021, applied qualitative methods with thematic analysis to written reflections and semi-structured interview transcripts discussing the implementation of C-CM in paramedic practice. A convenience-quota sample of 11 participants, consisting of third-year paramedic students and recent NQPs, were recruited. This research is reported using Consolidated Criteria for Reporting Qualitative Research (COREQ) reporting guidelines.

**Results:**

Eleven participants were recruited in total; nine consented to reflective writing analysis and interviews, two consented to writing analysis only. Analysis of the writing samples allowed for a deductive approach to the interview plan. Participants consisted of seven males and four females. All eleven participants (ten British and one Indian) spoke English as a first language. Ages ranged from 18 to 59 years. Career status was 46.2% third-year students and 53.8% NQPs. Four major themes were identified: barriers to implementation of the C-CM in practice; impact of C-CM on paramedic practice; C-CM as a teaching and learning tool in paramedic practice; and adaptation of C-CM for paramedic practice.

**Conclusions:**

Participants suggested that implementation of C-CM leads to improved structure and shared decision-making; however, adaptions to make it more paramedic-focused would be welcomed. The diversity of patients and their preferences can make its implementation challenging, and the negative feedback received from experienced ambulance staff was a significant concern to participants.

## Introduction

One of the most notable early authors in the field of patient consultation was Balint. His work emphasised progressing from paternalistic to patient-focused medicine, valuing patients’ experiences and opinions (Balint, 1957). Balint’s contributions marked the inception of patient-centred care (PCC), a concept that is now integral to NHS values (NHS England, 2021). Effective communication skills are crucial to establish a thorough assessment, especially concerning non-visible symptoms or key psychological or social details, for accurate working diagnoses and person-centred management (Manalastas et al., 2021). While acknowledging the necessity of swift assessment and treatment for time-critical emergencies, data from 2015/2016 reveals that 38% of individuals to whom an ambulance was dispatched were not subsequently transported to the hospital, indicating a substantial portion of cases handled by ambulances may not be time sensitive (O’Cathain et al., 2018). These patients have a need for thorough consultations to tailor appropriate, safe, patient-centred treatments at home or referrals to other services; therefore, providing student paramedics with education and tools to develop skills in this area should be considered essential. Research in this field is limited: a scoping review on undergraduate paramedic students and interpersonal communication development by Mangan et al. (2022) identified some current research, but none addressed the use of consultation models as a communication tool.

**Figure 1:**
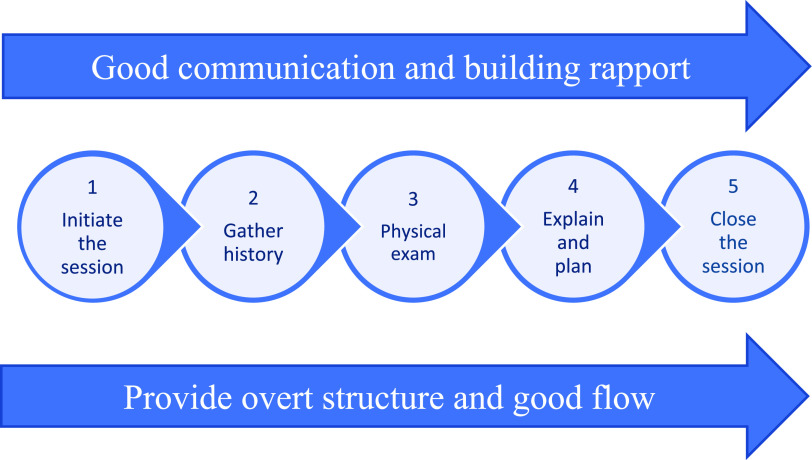
Figure 1. A summary of the main stages of the Calgary-Cambridge Model. There are five key stages to the consultation, indicated by the circles, with two overarching themes that run consistently throughout, indicated by the arrows above and below. Redrawn from Silverman et al. (2013).

Many people have built on the work of Balint, with numerus consultation models published, including the Byrne and Long Model (Byrne et al., 1976), Helman’s Folk model (Helman, 1981), The Consultation (Pendleton et al., 1984) and The Inner Consultation (Neighbour, 1987). The Calgary-Cambridge Model (C-CM) was first developed by Kurtz and Silverman (1996) and then enhanced by Kurtz et al. (2003) (see Figure 1 for summary of the model) and remains one of the most respected. While more recent data is unavailable, a 2009 cross-sectional survey of medical schools found that C-CM was used in 56% of them, indicating widespread popularity (Gillard et al., 2009). The C-CM was published with the intention of educating medical students, and it has been trialled or adopted by other professionals, such as pharmacists (Da Costa et al., 2020; Naughton, 2018) and nurses (Robinson et al., 2018). However, no published research on the use of C-CM by UK paramedics was found in the duration of this project. The author, a paramedic lecturer, affirms that consultation models are taught at four institutions where they have worked, with particular focus on C-CM, and contact with colleagues in other institutions suggests similar practice more widely. The College of Paramedic Education framework and Health and Care Professions Council standards of proficiency lack specific guidance on this practice, offering general advice on communication and history taking (HCPC, 2023; Hickson et al., 2019), and existing consultation models, designed for doctors to use in controlled settings, may not align with paramedics’ unpredictable environments.

Given the absence of evidence, this MSc dissertation project aimed to explore the experience of students and NQPs applying the C-CM in practice and establish their experiences and perceptions of its suitability as a communication tool in the pre-hospital environment.

## Methods

Conducting a consultation with a patient is considered by this researcher as a social interaction, and this research explores the participant experience and the participant’s perceived use of the C-CM within that interaction. Qualitative research methods will therefore be used to investigate the complexity of the behaviour, experiences and phenomena that occurs within these interactions (Green & Thorogood, 2018). A qualitative approach allows for depth of research to be prioritised over breadth to identify perspectives that may shape the actions of the individuals in the context of a patient consultation. Therefore, qualitative semi-structured interviews are used to produce data analysed by thematic analysis.

### Sampling

Due to an MSc assessment deadline, a convenience-quota sampling was chosen, efficiently using the university email system to recruit, after obtaining gatekeeper approval (Biggam, 2021). A participant information sheet and consent form were sent to student paramedics and NQPs at Sheffield Hallam University (SHU), trained in using C-CM, who had the opportunity to use it in practice. The participants were in their first 12 months of NQP or about to graduate within three months. This sample were from the first three cohorts to receive teaching and assessment on C-CM at SHU. Exclusions applied to third-year students who had not been successful in their assessment on C-CM. This form of sampling provided advantages, as variables could be limited and all participants were educated in C-CM using the same pedagogy, learned C-CM in year three of their programme, were educated in the same environment by the same core education team, and had practised the model within the previous 12 months.

A total of 192 potential participants were contacted, and 13 responses were received; however, two respondents did not confirm an interview date or provide access to their essay and so were withdrawn from the project. Low recruitment may be attributed to the timing of emails coinciding with student assessments, and graduated participants may not have checked their university email accounts. While 11 participants may seem to be a small sample group, qualitative research often finds richness in data with a limited number (Green & Thorogood, 2018; Lowe et al., 2018). Literature suggests varying opinions on the ideal participant count, ranging from five to fifty (Green & Thorogood, 2018). Hinton and Ryan (2020) suggest that for a phenomenological methodology ten participants is sufficient, with higher numbers reserved for grounded theory. All of these cited authors agree that ‘saturation’, where no new data emerges, indicates sufficient recruitment (Green & Thorogood, 2018; Hinton & Ryan, 2020; Lowe et al., 2018).

### Data collection

Data was collected between 1 April 2021 and 31 May 2021.

### Reflective essays

**Figure 2:**
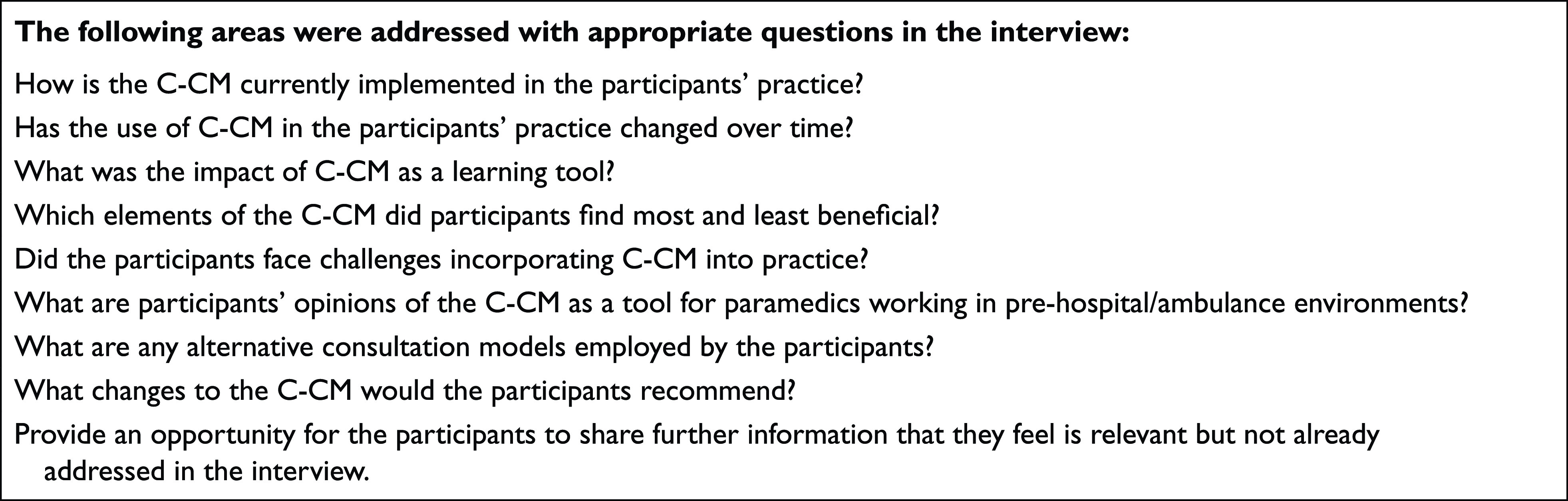
Figure 2. Interview guide.

Paramedic students wrote a 3000-word reflective account of the C-CM in practice in the last six months of their programme. In this essay, students were tasked with reflecting on their utilisation of the C-CM and critiquing its suitability for paramedic practice. The participants who consented allowed the researcher to analyse their anonymised reflective accounts. The analysis of essay data before the interviews afforded the opportunity to develop deductive themes for the interview guide for the subsequent semi-structured interviews (Braun & Clarke, 2022). The interview guide created for the semi-structured interviews can be seen Figure 2.

### Semi-structured interviews

Interviews were undertaken in addition to essay analysis to enhance the depth and richness of the data. Consenting participants joined video interviews from their own homes, a choice made for COVID-19 safety. Transcription software was used for efficient transcription within 24 hours. The one-to-one interviews prioritised a natural conversation without field notes; necessary notes were added during transcription verification with use of a recording.

Semi-structured interviews were chosen for their flexibility, enabling participants to freely respond to open-ended questions and allowing exploration of areas important to them outside the interview plan (Hinton & Ryan, 2020). Participants received the interview guide one week in advance to mitigate the researcher’s time constraints, as there was insufficient time for a secondary interview. This allowed participants to contemplate themes beforehand, enabling a comprehensive exploration of their thoughts in a single interview. Participants were given two weeks to share additional thoughts post interview, with one participant availing this option; extra comments were integrated into the transcript. Each participant received a copy of the completed analysis; feedback was invited but none was received.

A pilot interview with a paramedic colleague determined that 30 to 60 minutes was sufficient for the interviews. The data from this session, conducted for the purpose of testing time requirements, transcription software and the researcher’s interview skills, was excluded from analysis, as it did not meet inclusion criteria.

### Data analysis

Data analysis occurred simultaneously with data collection between 1 April 2021 and 31 May 2021. The essays and the interview transcripts were analysed using thematic analysis (TA), as instructed by Braun & Clarke (2006). There are six analysis steps to follow: familiarising yourself with your data; generating initial codes; searching for themes; reviewing themes; defining and naming themes; and producing the report. TA was chosen for its flexibility across epistemological positions and its suitability for novice researchers. Although there are other analysis techniques, Braun & Clarke (2006) advise mastering TA before attempting more complex approaches. Data analysis reached saturation at Paramedic 8’s interview (Hinton & Ryan, 2020).

### Reflexivity

Although every effort was made to conduct the research impartially without influencing the participants in anyway, an interview is a social interaction and this makes it inevitable that some influence may occur (Peddle, 2022). Acknowledging the areas where influences may occur can make the researcher more aware of them and limit their impact (Peddle, 2022).

It is acknowledged that the researcher may have unintentionally influenced the data collected because they were a paramedic/lecturer at the time of the study. The lecturer‒student relationships between interviewer and interviewee may have led to reluctance in honesty from the participants. To mitigate this, each interview began with a statement from the researcher declaring no allegiance with the C-CM and explaining the need for honest answers to complete valuable research. It was also made clear that taking part in the research would not affect the third-year students’ grades in any way.

The research stemmed from the investigator’s background in both teaching and clinical practice within the paramedic field. Despite maintaining a neutral stance on the efficacy of the C-CM, their decision to evaluate its application by participants was driven by a desire for a comprehensive assessment of the model. However, it is important to acknowledge that the researcher’s inherent subjectivity could impact the interpretation of the findings, as they serve a pivotal instrument in the analysis process. Thus, reflexivity should offer readers insight into the researcher’s perspective, thereby enhancing transparency. While a second researcher review may be considered ideal, this wasn’t feasible due to the assessment constraints of the MSc, and that should be taken into account when reading this article. The absence of qualitative software is noted, with the manual method chosen for its immersive benefits despite potential trustworthiness gains from software use (O’Kane et al., 2021).

## Results

Eleven participants were recruited; nine consented to reflective essay analysis and interview and two consented to reflective essay analysis only. The participants’ characteristics were collected to detail their diversity, with responses given in their own words and with an option to refrain from answering if preferred. This data can be seen in Table 1.

**Table 1.** Participant characteristics as described in participants’ own words.

Category	Diversity of participant group
**Age**	18–29 = 42.2% 30–39 = 38.5% 40–49 = 7.6% 50–59 = 7.7%
**Sex**	Male = 7 Female = 4
**Sexuality**	Heterosexual = 10 Lesbian = 1
**Ethnicity**	White British = 10 Indian = 1
**First language**	English = 11
**Disability**	Hearing deficit = 1 None declared = 10
**Career status**	Third-year student = 46.2% NQP = 53.8%

**Figure 3:**
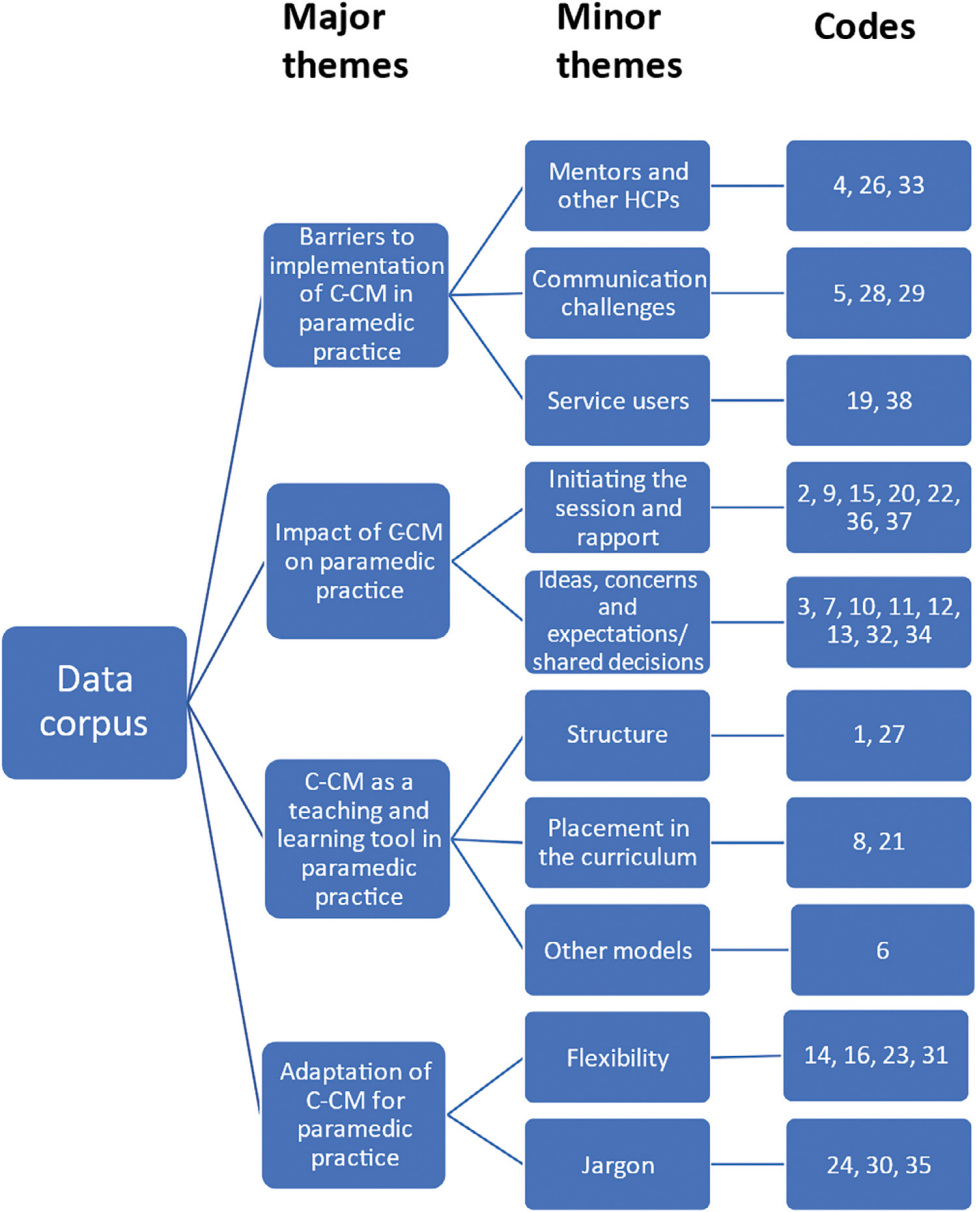
Figure 3. Diagram of the coding tree.

Completion of TA themes was established as illustrated in Figure 3, and the coding key can be seen in Table 2. Transcript extracts are included to support reliability (Braun & Clarke, 2022). Participants are denoted Paramedic 1‒11.

**Table 2.** Coding key.

Code	Participant discusses
**1**	Structure
**2**	Rapport
**3**	Shared decisions
**4**	Influence of other paramedics
**5**	Patients with communication difficulties
**6**	Other consultation models
**7**	Ideas, concerns and expectations
**8**	Placement of C-CM in curriculum
**9**	Initiating session
**10**	Gathering information
**11**	Physical examination
**12**	Explanation and planning
**13**	Closing session
**14**	Adapting the model
**15**	C-CM in current practice
**16**	Use with non-acute patients
**17**	Holistic care
**18**	Benefit to the NHS
**19**	Influence of patients on using the model
**20**	Other HCPs
**21**	C-CM as a learning tool
**22**	C-CM in previous practice
**23**	Use with acute patients
**24**	Jargon/terminology
**25**	Miscellaneous
**26**	NHS managers
**27**	Calgary-Cambridge Guide 70+ steps
**28**	Mental health/capacity
**29**	Children
**30**	Change of paramedic role
**31**	Suggested adaptations to the model for paramedics
**32**	Discharging patients at home
**33**	Does not use/dislikes model
**34**	Social history
**35**	Confidence
**36**	Chunks and checks
**37**	Trust
**38**	Time management

### Theme 1: barriers to implementation of the C-CM in practice

#### Mentors and other HCPs

Participants discussed instances of negative feedback from colleagues, including mentors or non-paramedic crew members, regarding their use of the C-CM, as illustrated below:

*I think staff are the biggest barriers. [. . .] Then they [staff members] ask you questions about what, why you’re doing that, what do you know, what you’re doing.* (Paramedic 9)

This comment is representative of a recurring pattern in the data set: experienced ambulance crew members observe consultations, then question participants about their approach. These interactions suggest a lack of familiarity with or perceived inappropriateness of the C-CM approach by some experienced crews, leading to a negative and non-constructive experience for participants.

#### Communication challenges

Participants highlighted challenges using C-CM with children, individuals with temporary cognitive issues due to alcohol or drug use, those with permanent cognitive deficits or non-English speakers. The model’s need for a detailed patient history through continued dialogue was seen as unachievable in some cases. Participants felt that the inability to fully use C-CM when there were communication obstacles impacted the quality of care provided.

#### Service users

Some participants perceived the service users themselves to be a barrier to using the model, and there were several examples given of patients not fully engaging in the consultation. Various reasons may explain a patient’s non-engagement with the model. While poor execution by the paramedics could contribute, the data suggests a preference for some patients to be told what to do by a healthcare professional, emphasising a desire for a more passive role in the process.

### Theme 2: impact of C-CM on paramedic practice

Participants expressed positive opinions about the impact of C-CM on their practice. They all believed that learning and applying the model had enhanced their practice, benefiting service users.

#### Initiating the session and building rapport

Opinion regarding this step of the C-CM was mixed; some suggested it helped their practice and others implied it was problematic. Here is an example of the former:

*[. . .] you know, you go in and introduce yourselves trying to, as soon as you can, start trying to sort of build that relationship [. . .] because if they don’t feel comfortable and they don’t trust you, they might not tell you something that could be, like, quite important.* (Paramedic 6)

In contrast, data in this minor theme also indicates that due to the unpredictable nature of ambulance attendance, the lack of patient notes and the unreliability of the information provided to the crew, building rapport was not a priority in the ‘initiating the session’ stage.

#### Ideas, concerns and expectations (ICE), and shared decision making

Silverman et al. (2013) recommend exploring ICE with the patient. This means the HCP understands early in the consultation the perspective of the patient and the desired outcome*.* It was typical of many of the participants to discuss the introduction of ICE into their practice; it encouraged them to achieve a better understanding of the patients’ perspective, resulting in a preferrable management outcome for the patient.

### Theme 3: C-CM as a teaching and learning tool in paramedic practice

#### Structure

Participants discussed the improvement in their consultation structure as a significant aspect of their learning. One paramedic’s quote exemplifies how using the model aided reflection and led to practice improvement.

*Yes, it’s definitely helpful. [. . .] Sometimes we miss something that once you’ve done it, you can go back and evaluate as well and get whatever you missed the first time.* (Paramedic 3)

#### Placement in the curriculum

C-CM was taught in the third year of study. All data concurred that introducing the model earlier in the programme would be preferable. Opinions varied on the appropriate stage for introducing the model, with some suggesting the first year and others proposing the second year.

#### Other models

A small number of participants recalled other consultation models introduced in the curriculum, suggesting a knowledge of several models can support paramedics to adapt their practice, as it provides additional tools from which to draw.

### Theme 4: adaptation of the C-CM for paramedic practice

Participants were in agreement that although applying the model was beneficial, adaptations for paramedic practice would be welcomed.

#### Flexibility

Participants noted that while they believed they covered all aspects of the model, they did not strictly follow the linear approach outlined by Silverman et al. (2013).

The dynamic nature of patients’ conditions that are often attended by ambulances requires an adaptable and fluid approach; a cyclical format instead of a linear flowchart was suggested:

*[. . .] like a circle to show that you know you can keep going back and asking the questions if you’ve missed sections [. . .] making it more kind of flowy and circular [. . .] I think with line straight down, it seems quite rigid, like you’ve got to follow it like this.* (Paramedic 6)

#### Jargon

Terminology varies across professions, and some paramedics found the C-CM to be doctor-centric, which is not surprising given its original design was for doctors. Several participants found the language off-putting, and believed that making it more paramedic focused could increase its popularity.

## Discussion

Ten minor themes were identified across the data, and these were grouped into four major themes, as presented in the results section. Theme 1 explores barriers faced by paramedics in implementing C-CM. Challenges include negative feedback from colleagues, communication difficulties with certain patient groups, and service users’ reluctance to actively contribute during consultations. In Theme 2, paramedics expressed positive opinions about the impact of C-CM on their practice, including the effectiveness of initiating sessions and building rapport, as well as the value of incorporating ICE into their practice. Theme 3 explores the C-CM as a teaching tool. Paramedics noted improvements in consultation structure and suggested introducing the model earlier in the curriculum. The advantages of an awareness of other consultation models were also discussed. In Theme 4, participants agreed on the benefits of the C-CM but suggested adaptations for paramedic practice. Flexibility was emphasised, with a preference for a cyclical approach rather than a linear one. Some paramedics found the terminology doctor-centric and proposed a more professional, yet paramedic-appropriate, language.

Broad searches of Medline, SCORPUS, CINAHL, Web of Science and Google Scholar at the time of writing failed to identify any research on the use of consultation models in paramedic practice. This makes comparisons from this current study challenging; however, three papers are of note, although they lack contemporaneity. Greenhill et al. (2011), in an observational study of pharmacists, evaluated C-CM in practice, indicating that the model was a useful tool to structure consultation with patients in pharmacies. Greenhill et al. (2011) suggested that adaptions be made by participants to make it more appropriate for pharmacists; adaptation of C-CM is also a key theme of this current study. Papageorgiou et al. (2011) studied the use of C-CM as a learning tool for medical students in a longitudinal consultation skills course. A cross-sectional survey conducted across each year of training revealed an overall increase in students’ confidence in consultation skills. Students in later years particularly valued the ongoing reinforcement of the model throughout their training. The results from the current study align with Papageorgiou et al. (2011); both disciplines value C-CM as a learning tool, with a suggestion that early introduction in an education programme is advantageous. Baniaghil et al. (2022) found, through a randomised control trial with midwives in Iran, that training using C-CM improved the skills of the students when consulting with patients. This was another positive example of the implementation of C-CM to alternative HCP students outside of medical schools.

Negative encounters with experienced crew members were identified as a prominent issue in the data from the current study. Unconstructive comments from mentors can impact the confidence of students/NQPs, reflecting typical hierarchical dynamics (Kim et al., 2020). Assertiveness training in paramedic curriculums may empower students to challenge mentors constructively (Omura et al., 2017). The mentor‒student relationship can be a valuable and rewarding one for both parties, and implementing improved support and training for mentors and qualified crew members could foster a more positive dialogue that promotes openness to students’ ideas and discourages poor hierarchical behaviour (Burgess et al., 2018).

Logical solutions for some of the communication barriers experienced by the participants could include sign language or Makaton; however, incorporating this into paramedic education could be challenging, and it may take significant investment and time before the effects are evident in practice. Interpreter services and NHS translation services are available for addressing hearing deficits and language barriers (NHS England, 2018), but the unpredictable nature of ambulance attendance can hinder timely access to these services. Emerging multimedia solutions, such as telehealth or translation apps, offer hope but are not yet widely adopted (Noack et al., 2021). It is unacceptable for those who do not speak English as a first language or those that communicate in other ways to receive sub-standard care due to these barriers. This contributes to health inequalities (Raleigh & Holmes, 2021), and practical solutions are needed to address this.

Participants identified improved shared decision-making (SDM) as a key advantage of the model. This aligns with the literature emphasising the importance of PCC, recognised in NHS constitutional values (NHS England, 2021). Practising PCC involves holistically considering the patient and collaborating on a care plan tailored to their needs (Santana et al., 2018). The participants acknowledged that using the C-CM encouraged them to listen to patients’ views, which informed their care decisions. Some literature suggests patient involvement in health decisions can empower patients, enhance satisfaction and improve compliance with care plans (NHS England, 2019). However, evidence to prove SDM improves patient outcome is contested (Pilnick, 2023).

**Figure 4:**
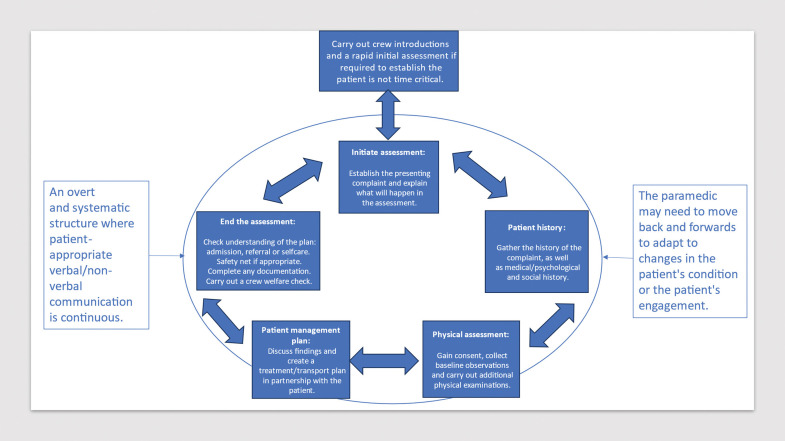
Figure 4. Adaptation of C-CM for paramedic practice and education. This diagram is a proposed adaptation of the original C-CM, and was created incorporating suggestions from the participants in the study. Note: further co-design and evaluation would be required to establish its suitability. Adapted from Silverman et al. (2013) by Claire Hastings.

Participants in this study suggested that using the C-CM helped create a structured patient assessment, but they also mentioned adapting the model to suit their needs. These adaptation patterns, also observed in graduate doctors by Manalastas et al. (2021), may indicate a response to patient behaviour, showing potential maturity in their approach. However, they could also suggest a lack of systematic work, risking omission of stages of the model. The data from this study, along with Manalastas et al. (2021), lacks sufficient detail to definitively answer this question. Participants in this current study suggested the model needs an additional step to determine patient acuity, followed by a cyclic flow through the existing five steps. Incorporating paramedic-focused terminology into the instructions could enhance the model for paramedic use further. Figure 4 has been produced to visually represent the adaptions to the model suggested in the data.

### Limitations and future research

While the limitations of substituting video call for face-to-face interviews should be considered, it is noteworthy that these participants, accustomed to extensive online learning and socialising during the Covid-19 pandemic, did not seem hindered by this. It was anticipated that participants being at home would create a calm environment but some may have experienced the opposite due to personal circumstances that the researcher was unaware of.

The convenience-quota sampling used in this study must be accounted for when considering the transferability of the results (Stratton, 2021), as it cannot be assumed that the perceptions and experiences of this group are representative of other student groups or of more experienced paramedics. Further interviews with paramedics who studied at alternative universities, in different areas of the country or with a more diverse population could add further nuance to the findings of this study. The study also acknowledges the limitations of a novice researcher and suggests a more experienced researcher might interpret the data differently.

Future research could explore how experienced paramedics approach low-acuity patient consultations, using ethnographic methods to identify consultation-style patterns. This may inform potential adaptations of the C-CM for ambulance practice or a new paramedic specific model. Co-producing and evaluating the practical application of an adapted C-CM would contribute to paramedic education and professional practice. Additionally, qualitative research is recommended to explore popular alternative consultation models in paramedic practice, after quantitative methods are used to identify them.

## Conclusion

This study has addressed the application of C-CM by student paramedics and NQPs, and was inspired by a gap in research on consultation models within paramedic practice. The findings reflect a nuanced landscape, identifying barriers to implementation, positive impacts on paramedic practice, its role as a teaching tool and the need for adaptations to suit paramedic-specific contexts. The positive impact on shared decision-making aligns with broader healthcare literature that emphasises the significance of PCC.

Recommendations from the analysis of the data include addressing negative encounters within hierarchical dynamics, incorporating adaptability into the model and refining the terminology to align with paramedic language contributing to optimising the utility of C-CM in paramedic practice. In conclusion, this study provides valuable insights into the challenges and benefits of implementing C-CM in paramedic practice. As paramedic education continues to evolve, considerations for effective consultation models are essential for fostering patient-centred, efficient and adaptable pre-hospital care.

## Acknowledgements

Michelle Rutherford (MSc, doctoral candidate) was the supervisor of the MSc project, and provided support and advice throughout the study but did not contribute to the authorship of this journal article. The author would like to extend thanks to Michelle Rutherford for her mentorship and encouragement. The author would also like to thank the participants for their time and co-operation with the project.

## Author contributions

CH was the lead researcher and author of this study. She designed the method and conducted all data collection, interviews and data coding. The study was conducted for her MSc dissertation. CH acts as the guarantor for this article.

## Conflict of interest

None declared. All participants were entered into a prize draw for a £20 gift voucher to thank them for their time.

## Ethics

Ethics approval was granted for the study by Glasgow Caledonian University (GCU) HLS PSWAHS Research Ethics Committee on 6 May 2021 with no conditions. Although the study was conducted with the co-operation of SHU students, the author was an MSc student at GCU at the time of the research.

## Funding

None.
